# Sugar-Sweetened Soft Drinks and Fructose Consumption Are Associated with Hyperuricemia: Cross-Sectional Analysis from the Brazilian Longitudinal Study of Adult Health (ELSA-Brasil)

**DOI:** 10.3390/nu10080981

**Published:** 2018-07-27

**Authors:** Jordana Herzog Siqueira, José Geraldo Mill, Gustavo Velasquez-Melendez, Alexandra Dias Moreira, Sandhi Maria Barreto, Isabela Martins Benseñor, Maria del Carmen Bisi Molina

**Affiliations:** 1 Centro de Ciências da Saúde, Universidade Federal do Espírito Santo, Vitória CEP 29042-755, Brazil; jordana.herzog@gmail.com (J.H.S.); josegmill@gmail.com (J.G.M.); 2 Escola de Enfermagem, Universidade Federal de Minas Gerais, Belo Horizonte CEP 30130-100, Brazil; jguveme@ufmg.br (G.V.-M.); alexandradm84@gmail.com (A.D.M.); 3 Hospital das Clinicas and School of Medicine, Universidade Federal de Minas Gerais, Belo Horizonte CEP 30130-100, Brazil; sandhi.barreto@gmail.com; 4 Clinical and Epidemiological Research Center, University Hospital, University of São Paulo, São Paulo CEP 05508-000, Brazil; isabensenor@hu.usp.br

**Keywords:** sugar-sweetened soft drinks intake, fruit and vegetable juices, fructose, uric acid, hyperuricemia

## Abstract

The secular trend of hyperuricemia coincides with the substantial increase in the consumption of sugar-sweetened beverages. Our aim was to evaluate the association between the consumption of soft drinks, dietary fructose and unsweetened, non-processed fruit juices with hyperuricemia in a cross-sectional analysis of baseline data (2008–2010; *n* = 7173) of the Brazilian Longitudinal Study of Adult Health (ELSA-Brasil). The explanatory variables were the consumption of soft drinks, fruit juice, and fructose using a validated semi-quantitative food frequency questionnaire. The outcomes were hyperuricemia and the uric acid concentration in serum. Regression models were tested, and a significance level of 5% was adopted. In men, the daily consumption of a portion of soft drink/day (250 mL) almost doubled the chance of hyperuricemia with a linear trend. In women, the consumption of ≥0.1 to <1.0 soft drink/day was associated with a higher chance of hyperuricemia, but there was no linear trend. High fructose consumption in men and moderate and high consumption in women were associated with hyperuricemia. All categories of soft drinks consumption were linearly associated with increased serum uric acid levels. Our findings suggest that the consumption of soft drinks and dietary fructose is positively associated with a higher chance of hyperuricemia and higher uric acid levels in Brazilian adults.

## 1. Introduction

A shift in dietary patterns has occurred in recent decades in several populations, including in Brazil, where a household survey reported an increase in the consumption of industrialized products [1]. Sugar-sweetened soft drink consumption increased 400% in Brazilian metropolitan areas from 1974–1975 to 2002–2003, corresponding to an increase of 0.4% to 2.1% of the total calories consumed daily [2]. According to the nationwide household survey (2008/09), the average consumption of these beverages in Brazilian adults was approximately 100 mL/day [3]. This scenario is worrying because the high consumption of soft drinks represents an important source of sugar, and it adds a significant amount of fructose to the diet. In addition, the high consumption of soft drinks is associated with an increased prevalence of weight gain, obesity and metabolic changes [4,5].

One of these changes is hyperuricemia, which is characterized by sustained elevation of serum uric acid levels [6], a factor responsible for gout, the most common inflammatory arthritis in men [7]. Increasing trends of hyperuricemia have been observed in several populations [8]. There is evidence of an association between hyperuricemia and metabolic [9] and cardiovascular diseases, such as arterial hypertension [10]. In Brazil, a population-based study in individuals aged 25 to 64 years found a hyperuricemia prevalence of 13.2% [11], which is lower than that observed in the United States, where a prevalence of 18% was reported in National Health and Nutrition Examination Survey III (NHANES) [6].

The consumption of meat, seafood, and alcohol has traditionally been considered an important risk factor for uric acid increase [12,13]. By contrast, dairy products, coffee, and foods rich in vitamin C exhibit protective action for hyperuricemia [14,15,16]. Although there is evidence that diet plays an important role in the development of hyperuricemia, the association with other dietary factors remains under investigation. Epidemiological studies in adult populations in the United States, Korea, and Mexico have found an association between the consumption of soft drinks and hyperuricemia [17,18,19]. However, the results are discordant in relation to fructose and fruit juices [6,14].

Studies of secular trends of increased hyperuricemia correlate with the increased consumption of sweetened beverages and fructose [8], which is supported by the role of fructose in lipid metabolism of the liver and of uric acid production [20]. Thus, uric acid production is positively regulated by fructose, unlike glucose and other monosaccharides [20]. Satiety and association with diseases do not differ with the type of sweetener used in the manufacturing of beverages, whether sucrose or corn syrup [21]. In Brazil, soft drinks are sweetened with sugar cane sucrose (50% glucose and 50% fructose) at a concentration of 8 to 11 g per 100 mL, whereas corn syrup (45% glucose and 55% fructose) has been used in the United States since 1967 with this same purpose [22,23].

The relationship between hyperuricemia, sweetened beverages and dietary fructose is not fully understood, and much of the research on sweetened beverage consumption and its health impact has focused on high-income countries. To date, no study has addressed this issue in the Brazilian population. Our objective was to verify whether there is an association between the consumption of sugar-sweetened soft drinks, fructose, and unsweetened beverages (natural fruit juices) and the prevalence of hyperuricemia in the participants of the Brazilian Longitudinal Study of Adult Health (ELSA-Brasil).

## 2. Materials and Methods 

This cross-sectional study was conducted with ELSA-Brasil baseline data (2008–2010). The cohort consisted of 15,105 active and retired civil servants aged 35–74 years old from six higher education institutions located in six Brazilian states (São Paulo, Minas Gerais, Bahia, Rio Grande do Sul, Rio de Janeiro, and Espírito Santo). The main objective of this study was to investigate the determinants and incidence of chronic diseases in the Brazilian population, with a primary focus on diabetes and cardiovascular diseases. The general characteristics of the cohort were previously published [24].

**Figure 1:**
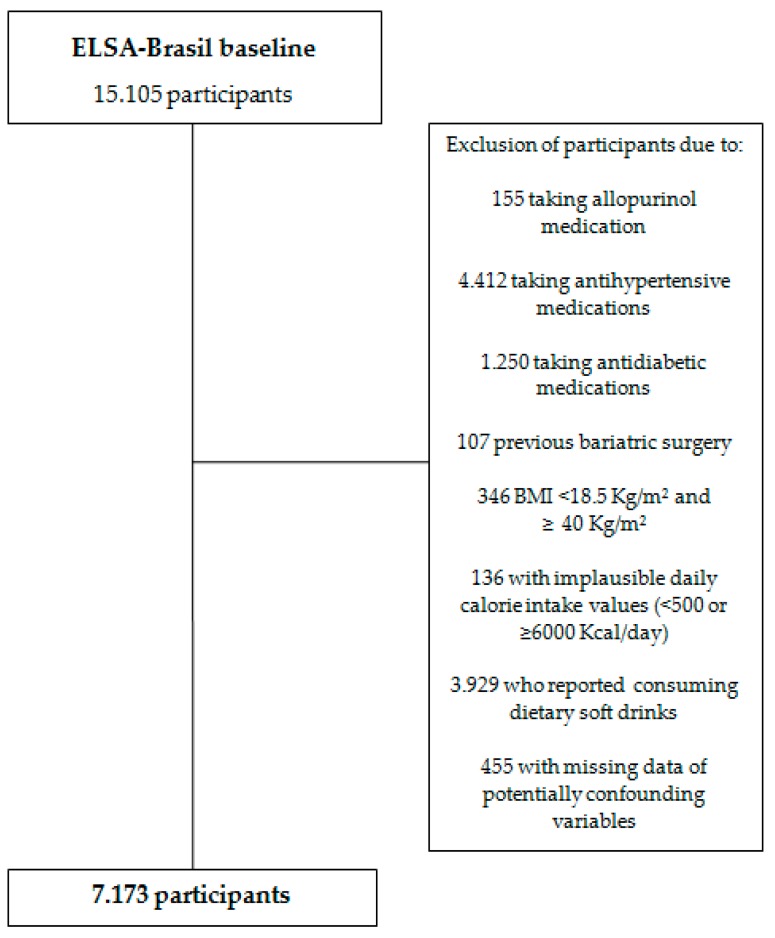
Exclusion flowchart for ELSA-Brasil participants.

Participants on a previously scheduled day attended the 7:00 am clinic for clinical, biochemical, and questionnaire exams. The biochemical tests were performed with 12-h fasting in all participants. In this way, a previously validated, comprehensive set of questionnaires, clinical measurements and laboratory tests was carried out. Food intake was investigated by a Food Frequency Questionnaire (FFQ). Socio-demographic data, self-reported health and life habits were also obtained during the interview. Body mass index (BMI) was calculated as body weight divided by height squared (kg/m²). We excluded from the present analysis the participants who have at least one of the following criteria: reporting use allopurinol, antihypertensive medications, antidiabetic medications, who had previously submitted to bariatric surgery, who presented an implausible daily caloric intake (<500 kcal/day or >6000 kcal/day), who consumed dietary soft drinks, with BMI < 18.5 kg/m² or ≥40 kg/m², and those with missing data of potentially confounding variables. The final sample consisted of 7123 participants (Figure 1).

General information on gauging and clinical exams can be found elsewhere [25]. Socio-demographic data (sex, age, income, and education) and lifestyle (smoking, physical activity, and alcohol consumption) were collected through a face-to-face interview using a standardized questionnaire [26]. Regarding the use of tobacco and alcohol, a questionnaire on the past and current use was implemented and the variables were coded in non-user, former and current.

Food intake was obtained through an FFQ that was developed and validated in the target population [27]. The FFQ of ELSA-Brasil is a semi-quantitative questionnaire composed of 114 items, whose objective is to evaluate habitual consumption in the last 12 months. The daily intake of nutrients and calories was estimated using the Nutrition Data System for Research (NDSR) software. The extreme values of consumption (above the 99th percentile) were replaced by the exact 99th percentile. In addition, when the participant voluntarily mentioned the seasonal consumption of some item/food or drink, the total amount of daily consumption of this food was multiplied by 0.25.

Regarding drinks, the participants were asked about their consumption (yes/no) and the frequency of consumption of a portion (equivalent to 250 mL). The participants were then grouped according to their daily intake of sugar-sweetened soft drinks: 0 portions/day, <0.1 portions/day, ≥0.1 to <1 portion/day and ≥1 portion/day. The same daily cut-off points were used for non-processed fruit juices. The total fructose in the diet was analysed in quartiles of consumption (g/day).

Blood samples were obtained when the participants were fasting and were quickly processed to obtain serum, which was stored locally at −80 °C until being sent to the Central Laboratory of ELSA-Brasil (São Paulo) monthly for the determination of analytes. The protocol of blood collection, processing and biological transport was standardized as described by Fedeli et al. (2013) [28]. Serum uric acid was determined by the Uricase (colorimetric enzyme) method (ADVIA 1200 automated analyzer—Siemens Healthcare Diagnostics, USA) and serum creatinine by the Jaffe method (ADVIA 1200 automated analyzer—Siemens Healthcare Diagnostics, USA), after the application of a conversion factor [29]. Hyperuricemia was defined as serum uric acid > 7.0 mg/dL in men and >6.0 mg/dL in women [30]. To assess renal function, the glomerular filtration rate (GFR) (mL/min/1.73 m²) was calculated according to the Chronic Kidney Disease Epidemiology Collaboration (CKD-EPI) equation [31], but no correction by race was implemented, according to validation studies for the Brazilian population [32,33].

Body measurements (weight, height and waist circumference [WC]) were measured according to standard procedures. The cut-off points used to assess WC were those recommended by the World Health Organization (WHO) [34]. Physical activity was estimated from the International Physical Activity Questionnaire (IPAQ) long version, in the domains of leisure physical activity in free time and physical activity of displacement [35], and was categorized as low, moderate, and high.

Potentially confounding variables were divided into the following categories: age (35–55, 45–54, 55–64, and 65–74 years), education (higher, average, fundamental), per capita income (in dollar), smoking (non-smoker, former smoker, and smoker), consumption of alcohol (never used, ex-user, and user), daily fruit consumption (yes/no), daily vegetable consumption (yes/no), use of vitamin supplements (yes/no), menopause (yes/no), hormone replacement therapy (yes/no), and WC (adequate/inadequate). The mean daily consumption of calories (quartis), meat and seafood (g/day), milk and dairy products (g/day), vitamin C (mg/day), and glomerular filtration rate (GFR) (mL/min/1.73 m²) was also assessed. WC was used as an adjustment instead of BMI because it is a commonly used method to assess visceral adiposity [36]. Thus, the accumulation of visceral fat, rather than subcutaneous fat, is associated with hyperuricemia in patients with primary gout [37].

Exploratory analyses by sex and evaluating the relationship between hyperuricemia and socio-demographic variables, life habits, self-reported health and food consumption were performed. Stratification by sex was justified by the presence of interaction on the association between outcome and exposure variables. To test the significant differences between the groups, we used Student’s *t*-tests for the continuous variables and the chi-squared test for the categorical variables. Sex-stratified multivariate logistic regression models were used to estimate the parameters of association between the consumption of sugar-sweetened soft drinks, fruit juices, dietary fructose, and hyperuricemia, after adjusting for potentially confounding variables (Odds Ratio [OR] and 95% confidence intervals [CI]). Model 1 included the sociodemographic variables age, sex, schooling, and per capita income. Model 2 included the variables of model 1, in addition to smoking, physical activity, alcohol use, WC, and GFR. Model 3 included the variables of models 1 and 2 in addition to caloric intake, daily consumption of fruits and vegetables, consumption of meat, fish and seafood, milk and dairy products, vitamin C intake, coffee consumption, and the use of vitamin supplements. For women, model 3 was further adjusted for menopause and the current use of hormone therapy.

Multiple linear regression models adjusted for the same covariates (models 1, 2, and 3) were also performed to verify the associations between the consumption of soft drinks, non-processed fruit juice and fructose and the serum levels of serum uric acid. In both models, a linear trend test (likelihood ratio test) was performed.

To isolate the effect of the addition of sucrose in sugar-sweetened soft drinks, which is responsible for metabolic effects, the analyses were repeated using unsweetened, non-processed fruit juice as the main exposure variable. Total dietary fructose (g/day) was also analysed.

The data were processed and analysed using Stata statistical software, version 12.0 (Stata Corp., College Station, TX, USA). The level of significance was set at *p* < 0.05. ELSA-Brasil was approved in the Research Ethics Committees of each participating institution. All participants signed an informed consent form.

## 3. Results

In this analysis, 7173 participants (46.4% men) from the ELSA-Brasil baseline were included; their general characteristics are described in Table 1. The mean age was 50 ± 8.4 years, and most participants had higher education. The mean serum uric acid level was 6.3 ± 1.3 mg/dL in men and 4.5 ± 1.0 mg/dL in women (*p* < 0.001). The prevalence of hyperuricemia was 17.3% (*n* = 1242), corresponding to 27.3% in males and 8.7% in females (*p* < 0.001). Most of the characteristics evaluated differed between men and women, including age, income, education, smoking, alcohol use, physical activity, WC, and vitamin/mineral supplement use. Men reported consuming an average of 45 ± 82 kcal/day from soft drinks, whereas women reported 22 ± 56 kcal/day (*p* < 0.001). The consumption of soft drinks was higher in males than in females (*p* < 0.001), whereas that of fruit juices was higher in females (*p* = 0.050). Other variables of food consumption, including daily consumption of fruits and vegetables, meat, fish and seafood, dairy products, fructose, and total daily calories, were also significantly different between the sexes.

Table 2 shows the demographic and clinical characteristics of men and women with normal or elevated uric acid levels. The frequency of hyperuricemia was relatively stable as age increased in men, but it was markedly increased in women. Alcohol consumption increased the frequency of hyperuricemia in men, whereas this habit hardly affected women. According to the dietary habit analysis, the consumption of fruits and vegetables was largely independent of the presence or absence of hyperuricemia. However, both men and women with hyperuricemia consumed smaller amounts of dairy products (*p* < 0.001 and *p* = 0.004, respectively). Higher consumption of meat, coffee, and sugar-sweetened soft drinks was also observed in men who had hyperuricemia (*p* = 0.005, *p* = 0.005 and *p* < 0.001, respectively), whereas women with the same condition had a higher consumption of fish and seafood (*p* = 0.004) and total daily fructose (*p* = 0.004).

The results of the multiple logistic regression analysis showed a positive association between sugar-sweetened soft drinks and fructose consumption and the presence of hyperuricemia in men (Table 3). In this group, all categories of sugar-sweetened soft drink consumption were associated with a higher probability of hyperuricemia compared to non-consumers. Even after adjustment for socio-demographic variables, life habits, health and food consumption, positive associations remained. After adjustment, the daily consumption of a portion of sugar-sweetened soft drinks (250 mL) almost doubled the occurrence of hyperuricemia (OR = 1.89, 95% CI, 1.39–2.57); there was also a dose-response gradient. In women, however, the consumption of ≥0.1 to <1.0 servings of sugar-sweetened soft drinks/day was associated with a higher chance of hyperuricemia (OR = 1.61, 95% CI, 1.18–2.18), but without a dose-response gradient. Regarding fructose, the highest quartile of consumption in men (OR = 1.30, 95% CI 1.00–1.68) and the third and fourth quartiles of consumption in women (OR = 1.48, 95% CI, 1.03–2.14; OR = 1.47, 95% CI, 1.00–2.20, respectively) were positively associated with the presence of hyperuricemia after adjustment. However, the dose-response was only observed in men. Conversely, the consumption of fruit juice was not associated with hyperuricemia.

The multivariate linear regression analysis results evaluating the effects of the consumption of sugar-sweetened soft drinks, fruit juice and fructose on serum uric acid levels are presented in Table 4. In the adjusted model, all categories of the consumption of soft drinks were associated with uric acid in both men and women. There was an average increase of 0.11 mg/dL of uric acid in men who consumed > 0 to <0.1 servings of sugar-sweetened soft drinks/day compared to non-consumers; (*p* < 0.001); in those who consumed >1.0 servings per day, the increase was 0.30 mg/dL (*p* < 0.001). In women with the same ranges, the mean increase was 0.08 mg/dL, 0.12 mg/dL and 0.15 mg/dL (*p* = 0.013), respectively. After adjusting for all confounding variables, the uric acid levels in men who were in the highest quartile of daily fructose consumption were shown to increase by an average of 0.20 mg/dL (*p* = 0.022). For women, no association was found. The consumption of fruit juice was not statistically associated with serum uric acid levels.

## 4. Discussion

According to our results, both men and women with a greater consumption of sugar-sweetened soft drinks had a greater chance of hyperuricemia compared to those who reported not consuming these drinks, even after adjusting for sociodemographic variables, life habits, self-reported health, and food consumption. In addition, the results show a dose-response gradient between sugar-sweetened soft drink consumption and hyperuricemia in men, a tendency that was not observed in women. The consumption of sugar-sweetened soft drinks was also linearly associated with serious uric acid levels in both men and women. In addition, the consumption of dietary fructose in the upper quartile was associated with uric acid levels and hyperuricemia in men with a dose-response gradient, whereas the third and fourth quartiles of consumption were associated with hyperuricemia in women, but without a dose-response gradient. By contrast, fruit juice was not associated with uric acid levels and a higher chance of hyperuricemia. According to our knowledge, this study is the first to report an association between sugar-sweetened soft drink intake, total dietary fructose, and hyperuricemia in a sample of the Brazilian population.

The positive association between sugar-sweetened soft drink consumption and increased serum uric acid levels is supported by several studies [6,17,18,19]. In the present study, the chance of hyperuricemia increased significantly with the ingestion of a portion/day of sugar-sweetened soft drinks, resulting in an 89% increase in the chance of developing hyperuricemia in men; in women, consuming ≥0.1 to <1 servings/day increased the change of hyperuricemia by 61% compared to non-consumers. A similar result was found in the Mexican population: the chance of hyperuricemia increased significantly by 129% in men with an intake of 3 portions/day of sugar-sweetened beverages and by 35% in women, compared to participants who consumed less than 0.5 servings/day of sugar-sweetened beverages [19]. Despite a study by Zgaga et al. (2012) [14] in Scotland that found a positive association between sweetened beverage consumption and serum uric acid, there was no association with fructose intake. This may be possible by the fact that fructose exerts beneficial effects at low doses and adverse effects only at higher doses [38]. However, our data corroborate with epidemiological studies conducted in the United States [6,39].

Differences in the prevalence of hyperuricemia by sex (27.3% in men and 8.7% in women) seem to be explained by the metabolic responses produced by female sex hormones. Studies show that oestrogen therapy in transsexual men reduces plasma uric acid and increases uric acid in urine, suggesting that oestrogen stimulates the renal clearance of urate [40]. Our results, which showed no dose-response gradient in the chance of hyperuricemia between the groups of soft drink intake and total fructose in women, can be explained by the relatively lower consumption of sugar-sweetened soft drinks and fructose among women compared to men.

The mean difference in serum uric acid levels between extreme categories of sugar-sweetened soft drinks consumption was estimated at 0.20 mg/dL in men, which is consistent with results from other studies [6,18]; moreover, it appears to support an association with prevalent hyperuricemia and a probable contribution to a risk of gout [41]. Prospective analyses have shown that elevations in serum uric acid from the consumption of sugar-sweetened soft drinks contribute to an increased risk of gout in men and women [6,40]. The association between the consumption of more than one portion of sugar-sweetened soft drinks per day and the chance of developing hyperuricemia was identified in the cross-sectional analysis [42], but the longitudinal analysis did not confirm these findings.

Fruits and fruit juices are considered a source of fructose, fibre, and antioxidants, such as carotenoids and vitamin C, the latter of which has a uricosuric effect [43]. A randomized clinical trial showed that 500 mg/day of vitamin C supplementation reduced serum uric acid levels by 1.5 mg/dL [44]. Although some studies have found an association between fruit juice and high uric acid levels [6,41], there was no greater chance of developing hyperuricemia with the increased intake of fruit juice in the present study. Similar results were reported by GAO et al. (2007) [17], who used data from the NHANES 2001–2002. This fact can be explained by the relatively low consumption among participants and the availability of vitamin C and fibre in fruit juice to compensate for the deleterious effects of fructose.

Studies on the implications of the type of sugar used to sweeten soft drinks (corn syrup or sucrose) have focused on the metabolism of fructose. Fructose is the only carbohydrate that has a direct effect on the metabolism of uric acid. There is strong evidence that fructose in the liver increases the degradation of adenosine triphosphate (ATP) to adenosine monophosphate (AMP), a precursor of uric acid. During its metabolism, phosphorylation by the enzyme fructose kinase results in fructose-1-phosphate (F1P). As a result, overloading this metabolic pathway may produce ATP depletion and inhibit adenosine diphosphate (ADP) phosphorylation, resulting in a lack of inorganic phosphate (Pi). Consequently, F1P is sequestered, and the ADP produced during this metabolic cycle is converted to AMP by adenylate kinase, which can also serve as a substrate for the production of uric acid. In addition, the depletion of ATP and Pi decreases feedback inhibition for uric acid generation. Experimental studies in humans and animals show a short-term increase in uric acid concentrations with the ingestion or perfusion of fructose [20,45]. Other metabolic abnormalities induced by fructose seem to stimulate the synthesis of long chain fatty acids and lead to hypertriglyceridemia and insulin resistance [46]. Both conditions have been associated with elevated serum uric acid levels [47].

The consumption of sugar-sweetened soft drinks in the country has increased considerably in recent decades. Therefore, these findings are important for public health since hyperuricemia has been associated with the development of various diseases and health conditions, such as hypertension, obesity, metabolic syndrome, and cardiovascular diseases [9,10,48]. A Brazilian study identified an average soft drinks consumption of 100 mL/day in adults [3], similar to that found in this study. The caloric contribution of soft drinks to the diet of ELSA-Brasil participants was estimated at 2% (data not shown in the table), according to survey data representative of the Brazilian population [49], which is less than that observed in the United States [50]. Notably, the increase in the consumption of soft drinks in Brazil can be attributed to the growth of the food processing industry in parallel with the expansion of supermarkets, [49] ineffective legislation and supervision, low marketing costs and extensive marketing [51]. Since the consumption of soft drinks represents a modifiable component of the diet and fructose is an independent risk factor for chronic diseases [52], a decrease in consumption may have a significant impact on reducing negative cardiometabolic outcomes.

Some limitations of this work deserve attention. The cross-sectional design limits the attribution of a causal relation of the associations due to a lack of temporality. However, the biological plausibility of the relationship between the high consumption of sugar-sweetened soft drinks, dietary fructose, and hyperuricemia, and, finally, the dose-response of this association, at least in men, was moderated by the low possibility of reverse causality (the participants were not aware of having hyperuricemia). In addition, the limitation of the instrument used (FFQ) for food consumption assessment is highlighted, due to the possibility of caloric overestimation. This problem is often reported, but it is possible to minimize it with greater quality control in data collection, as previously mentioned. In addition, participants who reported implausible ingestion values were also excluded. However, the methodological aspects of this study strengthen the validity of the results. Serum uric acid is a biological measure, and although there is no consensus regarding the cut-off values for hyperuricemia, the criterion adopted in this study approached the 90th percentile of the normal distribution curve. It is still important to note that in ELSA-Brasil all collection of biological material, processing, storage, and transport procedures were standardized. There was no loss of biological material due to transportation and no samples had to be discarded due to inadequate transportation. Thus, no factors affecting serum uric acid levels in this population were observed.

It is the first epidemiological study that identified the role of soft drinks consumption and dietary fructose in uric acid metabolism in the Brazilian population. Since the statistical analysis in our study was stratified by sex, it was possible to consider biological and lifestyle differences. It is important to highlight the exclusion of participants with type 2 diabetes and those who reported the consumption of diet soft drinks because they could have received medical advice and were advised to follow a restrictive sucrose diet. In this way, inclusion in the study could underestimate the results found. The standardization of routines and consolidated procedures for conducting the interview, measurement of anthropometric and biochemical measures, and periodic training in all research centres are also strengths of this study.

## 5. Conclusions

Our data showed that the consumption of sugar-sweetened soft drinks and dietary fructose may raise the serum uric acid level and increase the chance of developing hyperuricemia in Brazilian adults, although there was a discrepancy between a growing linear tendency and sex. The search for strategies to reduce or slow the expansion of the consumption of industrialized products today is essential since the high consumption of sugar-sweetened soft drinks is a public health problem in several populations, in addition to highlighting the necessity of routine dosage of serum uric acid in the clinical practice. New intersectoral health policies and strategies, including in the food and commerce industry, should discourage the consumption of such beverages and emphasize the adoption of a healthy lifestyle. We understand that measures are necessary at different levels, such as a tax increase on the volume of purchases of sugar-sweetened soft drinks and food and nutritional education actions. Finally, longitudinal studies with larger populations and in different ethnic groups are necessary to confirm the clinical significance of the association between the intake of sugar-sweetened soft drinks, dietary fructose, and hyperuricemia.

## Figures and Tables

**Table 1:** Baseline characteristics of participants according to sex. ELSA-Brasil, 2008–2010.

Variable	Male (*n* = 3.325)	Female (*n* = 3.848)	*p*-Value *
**Age (years)**			0.045
35 to 44	996 (30)	1064 (27.7)	
45 to 54	1418 (42.6)	1640 (46.2)	
55 to 64	707 (21.3)	912 (23.7)	
65 to 74	204 (6.1)	232 (6.0)	
**Race**			0.441
No white	1690 (50.8)	1948 (50.6)	
White	1635 (49.2)	1900 (49.4)	
**Education**			<0.001
Elementary	552 (16.6)	309(8.0)	
Middle and high school	1198 (36.0)	1447 (37.6)	
College or higher	1575 (47.4)	2092 (54.4)	
**Smoking**			<0.001
Non-smoker	1782 (53.6)	2442 (63.5)	
Former smoker	1009 (30.3)	887 (23.1)	
Smoker	534 (16.1)	519 (13.5)	
**Alcohol consumption**			<0.001
Non-drinker	145 (4.4)	551 (14.3)	
Former drinker	648 (19.5)	729 (18.9)	
Current drinker	2532 (76.2)	2568(66.7)	
**Physical activity during leisure time**			<0.001
Low	2476 (74.5)	3095 (80.4)	
Moderate	481 (14.5)	454 (11.8)	
High	368 (11.1)	299 (7.8)	
**Waist circumference**			<0.001
Adequate	1949 (58.6)	1447 (37.6)	
Inadequate	1376 (41.4)	2401 (62.4)	
**Hyperuricemia**			<0.001
Yes	907 (27.3)	335 (8.7)	
No	2418(72.7)	3513 (91.3)	
**GFR (mL/min/1.73 m²)**			0.060
<60	97 9 (2.92)	86 (2.23)	
≥60	3228 (97.08)	3762 (97.77)	
**Daily intake of fruits**			<0.001
Yes	1477 (43.5)	2371 (61.6)	
No	1878 (56.5)	1477 (38.4)	
**Daily intake of vegetables**			<0.001
Yes	1437 (43.2)	2116 (55)	
No	1888 (56.8)	1732 (45)	
**Use of vitamin supplements**			<0.001
Yes	285 (8.6)	550 (14.3)	
No	3040 (91.4)	3298 (85.7)	
**Age (years)**	49 ± 8.5	50 ± 8.3	0.035
**Per capita income (US$)**	1745 ± 2276	3136 ± 2633	<0.001
**Uric acid (mg/dL)**	6.3 ± 1.3	4.5 ± 1.0	<0.001
**GFR (mL/min/1.73 m²)**	86 ± 14	89 ± 15	<0.001
**Waist circumference (cm)**	92 ± 10	84 ± 11	<0.001
**Dietary intake**			
Meat intake (g/day)	205 ± 132	157 ± 115	<0.001
Seafood intake (g/day)	57 ± 66	50 ± 60	<0.001
Dairy food intake (g/day)	293 ± 278	340 ± 279	<0.001
Coffee intake (mL/day)	146 ± 127	145 ± 125	0.847
Sugar-sweetened soft drinks (mL/day)	117 ± 212	58 ± 145	<0.001
Fruit juice (mL/day)	44 ± 114	50 ± 119	0.050
Total fructose (g/day)	35 ± 20	32 ± 18	<0.001
Vitamin C (mg/day)	351 ± 250	349 ± 239	0.707
Total energy (kcal/day)	2647 ± 814	2123 ± 710	<0.001

GFR = Glomerular filtration rate. Data were expressed as mean ± SD for continuous variables and n (%) for categorical variables. * *p* < 0.05. Calculated by Student’s *t*-tests for continuous variables and chi-square tests for categorical variables.

**Table 2:** Characteristics according to hyperuricemia status. ELSA-Brasil, 2008–2010.

	Male (*n* = 3.325)			Female (*n* = 3.848)		
	Hyperuricemia			Hyperuricemia		
**Variable**	No	Yes	*p*-Value *	No	Yes	*p*-Value *
	(*n* = 2418)	(*n* = 907)		(*n* = 3513)	(*n* = 335)	
**Age (years)**			0.101			<0.001
35 to 44	744 (74.7)	252 (25.3)		1014 (95.3)	50 (4.7)	
45 to 54	1003 (70.7)	415 (29.3)		1507 (91.9)	133 (8.1)	
55 to 64	515 (72.8)	192 (27.2)		796 (87.3)	116 (12.7)	
65 to 74	156 (76.5)	48 (23.5)		196 (84.5)	36 (15.5)	
**Race**			0.311			0.385
No white	1242 (73.5)	448 (26.5)		1786 (91.7)	162 (8.3)	
White	1176 (71.9)	459 (28.1)		1727 (90.9)	173 (9.1)	
**Education**			0.005			<0.001
Elementary	383 (69.4)	169 (30.6)		265 (85.8)	44 (14.2)	
Middle and high school	862 (72.0)	336 (28.0)		1320 (91.2)	127 (8.8)	
College or higher	1173 (74.5)	402 (25.5)		1928 (92.9)	164 (7.8)	
**Smoking**			<0.001			<0.001
Non-smoker	1340 (75.2)	442 (24.8)		2267 (92.8)	175 (7.2)	
Former smoker	677 (67.1)	332 (32.9)		778 (87.7)	109 (12.3)	
Smoker	401 (75.1)	133 (24.9)		468 (90.2)	51 (9.8)	
**Alcohol consumption**			<0.001			0.885
Non-drinker	121 (83.4)	24 (16.6)		506 (91.8)	45 (8.2)	
Former drinker	510 (78.7)	138 (21.3)		663 (90.9)	66 (9.1)	
Current drinker	1787 (70.6)	745 (29.4)		2344 (91.3)	224 (8.7)	
**Physical activity during leisure time**			0.128			0.934
Low	1779 (71.8)	697 (28.2)		2823 (91.2)	272 (8.8)	
Moderate	358 (74.4)	123 (25.6)		416 (91.6)	38 (8.4)	
High	281 (76.4)	87 (23.6)		274 (91.6)	25 (8.4)	
**Waist circumference**			<0.001			<0.001
Adequate	1573 (80.7)	376 (19.3)		1405 (97.1)	42 (2.9)	
Inadequate	845 (61.4)	531 (38.6)		2108 (87.8)	293 (12.2)	
**GFR (mL/min/1.73 m²)**			<0.001			<0.001
<60	43 (44.3)	54 (55.7)		58 (67.4)	28 (32.6)	
≥60	2375 (73.6)	853 (26.4)		3455 (91.8)	307 (8.2)	
**Daily intake of fruits**			0.104			0.118
Yes	1073 (74.2)	374 (25.8)		2154 (90.8)	217 (9.2)	
No	1345 (71.6)	533 (28.4)		1359 (92.0)	118 (8.0)	
**Daily intake of vegetables**			0.479			0.023
Yes	1036 (72.1)	401 (27.9)		1914 (90.5)	202 (9.5)	
No	1382 (73.2)	506 (26.8)		1599 (92.3)	133 (7.7)	
**Use of vitamin supplements**			0.224			0.042
Yes	216 (75.8)	89 (24.2)		513 (93.3)	37 (6.7)	
No	2202 (72.4)	838 (27.6)		3000 (91.0)	298 (9.0)	
**Age (years)**	49 ± 9	50 ± 8	0.654	49 ± 8	53 ± 8	<0.001
**Per capita income (US$)**	2803 ± 2324	2584 ± 2137	0.014	3159 ± 2633	2904 ± 2632	0.089
**Uric acid (mg/dL)**	5.6 ± 0.8	8 ± 0.9	<0.001	4.3 ± 0.8	6.6 ± 0.6	<0.001
**GFR (mL/min/1.73 m²)**	88 ± 14	81 ± 15	<0.001	89 ± 14	80 ± 15	<0.001
**Waist circumference (cm)**	90 ± 10	97 ± 10	<0.001	83 ± 10	94 ± 11	<0.001
**Dietary intake**						
Meat intake (g/day)	201 ± 132	215 ± 131	0.005	168 ± 116	165 ± 107	0.751
Seafood intake (g/day)	57 ± 66	59 ± 65	0.716	49 ± 58	59 ± 71	0.004
Dairy food intake (g/day)	306 ± 281	256 ± 264	<0.001	344 ± 279	298 ± 265	0.004
Coffee intake (mL/day)	142 ± 125	156 ± 130	0.005	145 ± 126	146 ± 119	0.942
Sugar-sweetened soft drinks (mL/day)	110 ± 205	140 ± 230	<0.001	57 ± 146	63 ± 130	0.502
Fruit juice (mL/day)	44 ± 116	41 ± 107	0.464	49 ± 119	46 ± 116	0.593
Total fructose (g/day)	34 ± 19	35 ± 21	0.989	32 ± 17	36 ± 20	0.001
Vitamin C (mg/day)	348 ± 245	358 ± 260	0.293	348 ± 239	353 ± 229	0.742
Total energy (kcal/day)	2643 ± 810	2658 ± 826	0.626	2112 ± 706	2128 ± 743	0.909

GFR = Glomerular filtration rate. Data were expressed as mean ± SD for continuous variables and n (%) for categorical variables. * *p* < 0.05. Calculated by Student’s *t*-tests for continuous variables and chi-square tests for categorical variables.

**Table 3:** Multivariate-adjusted ORs (95% CIs) of hyperuricemia according to categories of intake of sugar-sweetened soft drink, fruit juice, and dietary fructose. ELSA-Brasil, 2008–2010.

Variable	*n*	Model 1	Model 2	Model 3
		OR (95% CI)	OR (95% CI)	OR (95% CI)
**Male (*n* = 3.325)**				
**Sugar-sweetened soft drinks (servings/day)**				
0	933	1.00	1.00	1.00
>0 to<0,1	666	1.26 (1.00–1.60)	1.21 (1.00–1.55)	1.25 (1.00–1.60)
≥0.1 to<1	1382	1.60 (1.31–1.96)	1.54 (1.25–1.90)	1.62 (1.30–2.01)
≥1	344	1.84 (1.39–2.43)	1.74 (1.30–2.34)	1.89 (1.39–2.57)
***p*** *		<0.001	<0.001	<0.001
**Fruit juice (servings/day)**				
0	2.506	1.00	1.00	1.00
>0 to<0.1	187	0.89 (0.62–1.27)	0.86 (0.60–1.25)	0.86 (0.59–1.25)
≥0.1 to ≤0.9	498	1.13 (0.90–1.41)	1.17 (0.93–1.48)	1.24 (0.97–1.57)
≥1	134	1.09 (0.73–1.62)	1.08 (0.71–1.64)	1.29 (0.84–1.98)
***p*** *		0.604	0.452	0.441
**Total fructose (g/day)**				
Quartile 1 (14 ± 4)	805	1.00	1.00	1.00
Quartile 2 (25 ± 3)	774	0.82 (0.65–1.03)	0.81 (0.64–1.02)	0.84 (0.66–1.07)
Quartile 3 (36 ± 4)	835	0.77 (0.62–0.96)	0.78 (0.62–0.98)	0.86 (0.67–1.11)
Quartile 4 (60 ± 17)	911	1.04 (0.85–1.29)	1.11 (0.89–1.39)	1.30 (1.00–1.68)
***p*** *		0.005	0.004	0.002
Female (*n* = 3.848) **				
**Sugar-sweetened soft drinks (servings/day)**				
0	1660	1.00	1.00	1.00
>0 to <0.1	1083	1.08 (0.80–1.44)	1.01 (0.74–1.37)	1.00 (0.74–1.17)
≥0.1 to <1	947	1.70 (1.28–2.26)	1.59 (1.18–2.13)	1.61 (1.18–2.18)
≥1	158	1.34 (0.74–2.41)	1.16 (0.63–2.13)	1.14 (0.61–2.13)
***p*** *		0.142	0.090	0.080
**Fruit juice (servings/day)**				
0	2747	1.00	1.00	1.00
>0 to <0.1	287	0.73 (0.44–1.20)	0.77 (0.46–1.28)	0.80 (0.48–1.33)
≥0.1 to <1	666	0.81 (0.58–1.12)	0.84 (0.60–1.17)	0.87 (0.61–1.22)
≥1	148	1.00 (0.55–1.18)	0.96 (0.52–1.77)	0.91 (0.49–1.70)
***p*** *		0.442	0.634	0.717
**Total fructose (g/day)**				
Quartile 1 (15 ± 4)	988	1.00	1.00	1.00
Quartile 2 (26 ± 3)	1019	1.21 (0.86–1.71)	1.26 (0.89–1.79)	1.32 (0.92–1.89)
Quartile 3 (35 ± 4)	959	1.42 (1.01–1.98)	1.43 (1.01–2.02)	1.48 (1.03–2.14)
Quartile 4 (60 ± 16)	882	1.48 (1.05–2.06)	1.41 (1.00–2.00)	1.47 (1.00–2.20)
***p*** *		0.786	0.584	0.470

Portion = 250 mL/day. 95% CI = 95% confidence interval; OR = odds ratio. Model 1: Adjusted for years, education and per capita income; Model 2: Adjusted for years, education, per capita income, smoking, physical activity, alcohol consumption, glomerular filtration rate and waist circumference; Model 3: Adjusted for years, education, per capita income, smoking, physical activity, alcohol consumption, glomerular filtration rate, waist circumference, total energy, meat intake, seafood intake, coffee intake, dairy food intake, daily intake of fruits, daily intake of vegetables, use of vitamin supplements, Vitamin C, and another beverages in the table; * *p* for linear trend. ** For women, model 3 was additionally adjusted for menopause and postmenopausal hormone use.

**Table 4:** Linear regression coefficients (95% CIs) between serum uric acid levels and sugar-sweetened soft drinks intake, fruit juice intake and total fructose. ELSA-Brasil, 2008–2010.

Variable	*n*	Model 1	Model 2	Model 3
		β (95% CI)	β (95% CI)	β (95% CI)
**Male (*n* = 3.325)**				
**Sugar-sweetened soft drinks (servings/day)**				
0	933	0.00	0.00	0.00
>0 to <0.1	666	0.15 (0.02–0.28)	0.10 (−0.01–0.22)	0.11 (0.00–0.23)
≥0.1 to <1	1382	0.35 (0.24–0.46)	0.27 (0.17–0.38)	0.29 (0.18–0.39)
≥1	344	0.39 (0.22–0.55)	0.28 (0.13–0.44)	0.30 (0.15–0.46)
***p*** *		<0.001	<0.001	<0.001
**Fruit juice (servings/day)**				
0	2.506	0.00	0.00	0.00
>0 to <0.1	187	−0.00 (−0.20–0.19)	−0.06 (−0.18–0.17)	0.01 (−0.17–0.19)
≥0.1 to <1	498	0.00 (−0.12–0.13)	0.03 (−0.08–0.15)	0.06 (−0.05–0.18)
≥1	134	0.03 (−0.19–0.26)	0.04 (−0.17–0.25)	0.14 (0.06–0.35)
***p*** *		0.970	0.967	0.948
**Total fructose (g/day)**				
Quartile 1 (14 ± 4)	805	0.00	0.00	0.00
Quartile 2 (25 ± 3)	774	−0.01 (−0.14–0.11)	−0.00 (−0.12–0.11)	0.01 (−0.11–0.13)
Quartile 3 (36 ± 4)	835	−0.03 (−0.15–0.09)	0.01 (−0.10–0.12)	0.06 (−0.06–0.18)
Quartile 4 (60 ± 17)	911	0.08 (−0.04–0.20)	0.10 (−0.00–0.22)	0.20 (0.05–0.32)
***p*** *		0.300	0.413	0.022
Female (*n* = 3.848) **				
**Sugar-sweetened soft drinks (servings/day)**				
0	1660	0.00	0.00	0.00
>0 to <0.1	1083	0.11 (0.03–0.19)	0.08 (0.01–0.16)	0.08 (0.00–0.15)
≥0.1 to <1	947	0.19 (0.11–0.27)	0.13 (0.05–0.21)	0.12 (0.04–0.20)
≥1	158	0.28 (0.12–0.45)	0.16 (0.00–0.31)	0.15 (0.00–0.31)
***p*** *		<0.001	0.012	0.013
**Fruit juice (servings/day)**				
0	2747	0.00	0.00	0.00
>0 to <0.1	287	−0.06 (−0.16–0.03)	−0.00 (−0.09–0.08)	−0.06 (−0.18–0.04)
≥0.1 to <1	666	−0.07 (0.14–0.00)	−0.03 (−0.10–0.03)	−0.07 (−0.15–0.00)
≥1	148	−0.05 (−0.16–0.05)	−0.02 (−0.13–0.07)	−0.04 (−0.190.11)
***p*** *		0.235	0.519	0.488
**Total fructose (g/day)**				
Quartile 1 (15 ± 4)	988	0.00	0.00	0.00
Quartile 2 (26 ± 3)	1019	0.01 (−0.07–0.10)	0.00 (−0.07–0.09)	0.01 (−0.06–0.10)
Quartile 3 (35 ± 4)	959	0.06 (−0.02–0.15)	0.04 (−0.04–0.12)	0.05 (−0.02–0.14)
Quartile 4 (60 ± 16)	882	0.07 (−0.01–0.17)	0.03 (−0.05–0.11)	0.04 (−0.06–0.13)
***p*** *		0.873	0.806	0.667

Portion = 250 mL/day. 95% CI = 95% confidence interval; β = linear regression coefficient. Model 1: Adjusted for years, education and per capita income; Model 2: Adjusted for years, education, per capita income, smoking, physical activity, alcohol consumption, glomerular filtration rate and waist circumference; Model 3: Adjusted for years, education, per capita income, smoking, physical activity, alcohol consumption, glomerular filtration rate, waist circumference, total energy, meat intake, seafood intake, coffee intake, dairy food intake, daily intake of fruits, daily intake of vegetables, use of vitamin supplements, Vitamin C, and another beverages in the table; * *p* for linear trend. ** For women, model 3 was additionally adjusted for menopause and postmenopausal hormone use.
